# Carriage of *Staphylococcus aureus* among Portuguese nursing students: A longitudinal cohort study over four years of education

**DOI:** 10.1371/journal.pone.0188855

**Published:** 2017-11-30

**Authors:** Teresa Conceição, Hermínia de Lencastre, Marta Aires-de-Sousa

**Affiliations:** 1 Laboratory of Molecular Genetics, Instituto de Tecnologia Química e Biológica António Xavier, Universidade Nova de Lisboa, Oeiras, Portugal; 2 Laboratory of Microbiology and Infectious Diseases, The Rockefeller University, New York, NY, United States of America; 3 Escola Superior de Saúde da Cruz Vermelha Portuguesa, Lisboa, Portugal; Universitatsklinikum Munster, GERMANY

## Abstract

**Background:**

*Staphylococcus aureus* is a major human pathogen that can colonize healthy people mainly in the anterior nares. The aim of the present study was to evaluate *S*. *aureus* nasal colonization over time among Portuguese nursing students, including methicillin-resistant *S*. *aureus* (MRSA).

**Methods and findings:**

In this longitudinal cohort study, we collected 280 nasal swabs from nursing students at 14 time points over four years of schooling (2012–2016). The isolates were characterized by pulsed-field gel electrophoresis (PFGE), *spa* typing, multilocus sequence typing (MLST), and SCC*mec* typing for MRSA.

Among 47 students, 20 (43%) carried methicillin-susceptible *S*. *aureus* (MSSA) at admission, but none was colonized with MRSA. A total of 19 students (40%) became colonized after exposure during the nursing training, out of which five carried MRSA. Overall, 39 students (83%) had *S*. *aureus* detected at least once during the study period. Among the 97 MSSA isolates, most (65%) belonged to four clones: PFGE A-ST30 (21%), B-ST72 (20%), C-ST508 (13%), and D-ST398 (11%). Three of the five MRSA carriers were colonized with the predominant clone circulating in Portuguese hospitals (ST22-IVh) and two with ST3162-II. Colonization of nursing students was highly dynamic with continuous appearance of strains with distinct PFGE types in the same individual.

**Conclusions:**

A considerable proportion of students became colonized by *S*. *aureus*, including MRSA, during the nursing education, evidencing this population represents an important reservoir of *S*. *aureus*. Therefore, education on infection control measures in nursing schools is of major importance.

## Introduction

*Staphylococcus aureus* is a major human pathogen that can colonize healthy people. The anterior nares are the main ecological niche in humans, although the bacterium may be isolated also from multiple sites of the skin and mucosal surfaces [1]. The mean nasal carriage rate of *S*. *aureus* in the general population was estimated as 37% although different carrier states can be distinguished: (i) non-carriers, who are never colonized; (ii) persistent carriers, who are chronically colonized and frequently with the same strain, and (iii) intermittent carriers, who are colonized with different strains for short time periods [2].

According to a review on methicillin-resistant *S*. *aureus* (MRSA) nasal carriage among healthcare workers (HCWs), the reported carriage rate was 4.6% [3]. Noteworthy, nurses have a two-fold increased risk of being colonized with MRSA comparing with medical doctors and a three-fold higher risk than other HCWs [4]. It was shown previously that nurses have a better compliance with hand hygiene and infection control protocols [5], though the higher colonization rate may be due to closer and more frequent contact between nurses and patients and to the higher frequency of skin conditions in nursing staff, which has been previously demonstrated to be associated with MRSA acquisition [6]. Therefore, HCWs, with particularly nurses, may play an important role in MRSA transmission [7]. Although several studies evaluated the prevalence of *S*. *aureus* and MRSA among medical students, showing carriage rates of 14–45% and 0–5.4%, respectively [8], few studies focused on nursing students [9–11]. Moreover, most of the studies were cross-sectional and only a minority evaluated the carriage rates in different phases of the medical or nursing education, i.e. pre-clinical and throughout clinical exposure. Studies focusing on the MRSA nasal colonization among nursing students from Portugal, a country with a very high prevalence of MRSA (47%) [12], have been completely absent. Although Portugal is one of the European countries with the highest prevalence of MRSA among nosocomial invasive isolates [12], the prevalence of MRSA among the general population is very low; 1.8% among the elderly and <1% among young children [13,14].

The aim of the present study was to evaluate the dynamics of *S*. *aureus* and MRSA nasal colonization among nursing students over the four years of university attendance, including pre-clinical exposure and at different moments during clinical rotations. Moreover, we characterized the isolates for antimicrobial susceptibility, population structure and presence of the Panton-Valentine leukocidin (PVL) toxin among the *S*. *aureus* isolates.

## Material and methods

### Sampling and MRSA identification

In a longitudinal cohort study, we collected 280 nasal swabs from 47 nursing students in 14 consecutive screenings over four years of schooling (2012–2016) at Escola Superior de Saúde da Cruz Vermelha Portuguesa (ESSCVP), Lisbon, Portugal. The study was explained orally in the classroom to all nursing students admitted in September 2012. All individuals that accepted to participate (47 out of 55; rejection rate of 8.5%) were previously informed of the screening dates. Sampling and filling of the questionnaires were performed in the classroom.

After overnight enrichment growth at 37°C in Mueller-Hinton broth (Becton, Dickinson & Co, New Jersey, USA), the samples were inoculated on Tryptic Soy Agar (TSA) (Becton, Dickinson & Co, New Jersey, USA) and on chromogenic selective media Chromagar Staph aureus and Chromagar MRSA (ChromAgar, Paris, France). MRSA was confirmed by PCR amplification of the *nuc* and *spa* genes for species identification [15], and the detection of the *mecA* gene for methicillin resistance [16]. Informed consent was obtained from each participant. Demographic data, chronic disease, and risk factors for MRSA colonization were collected through questionnaires at each sampling.

Nasal colonization dynamics was evaluated considering the number of *S*. *aureus* acquisitions over the four-years period defined by (i) a change of colonization status from culture negative to culture positive or (ii) by a change of clonal type determined by PFGE type or subtype.

### Ethics statement

The protocol was approved by the Research Board of Escola Superior de Saúde da Cruz Vermelha Portuguesa. A verbal informed consent was obtained from each participant (all aged over 18) at the time of nasal screenings.

### Phenotypic and genotypic characterization

Antimicrobial susceptibility testing was performed by the disk diffusion method, according to the European Committee on Antimicrobial Susceptibility Testing (EUCAST_ http://www.eucast.org/), for cefoxitin, ciprofloxacin, chloramphenicol, clindamycin, erythromycin, fusidic acid, gentamicin, linezolid, mupirocin, oxacillin, penicillin, quinupristin-dalfopristin (Q-D), rifampin, teicoplanin, tetracycline, trimethoprim-sulfamethoxazole and vancomycin. These included all clinically relevant antibiotics. Vancomycin and linezolid are the two mostly used antibiotics for the treatment of MRSA in Portugal while all others are used for the treatment of MSSA, with the exception of quinupristin-dalfopristin that is not prescribed in the country. Oxacillin is clinically replaced by flucloxacillin.

The isolates were characterized by *spa* typing, multilocus sequence typing (MLST), SCC*mec* typing and by detection of the PVL gene as previously described [17].

### Statistical analysis

The sample size (n = 55) was calculated considering a margin of error of 10% and a confidence level of 95%, using the formula $n=\frac{\hat{p}\hat{q}}{\frac{B^{2}}{z^{2}}}$, where n is the sample size, Z is the selected critical value of desired confidence level, p is the estimated proportion of an attribute that is present in the population, q = 1-p, and B is the desired level of precision.

Risk factors were assessed using SPSS software, version 21.0. The chi-squared test was used to identify variables associated with *S*. *aureus* carriage (gender, previous hospital admission, previous surgery, current or previous antibiotherapy, chronic disease, smoking, contact with animals, household member working in a healthcare facility or nursing home) or the t-test (number of household members). P values of ≤0.05 were considered statistically significant.

## Results

**Fig 1:**
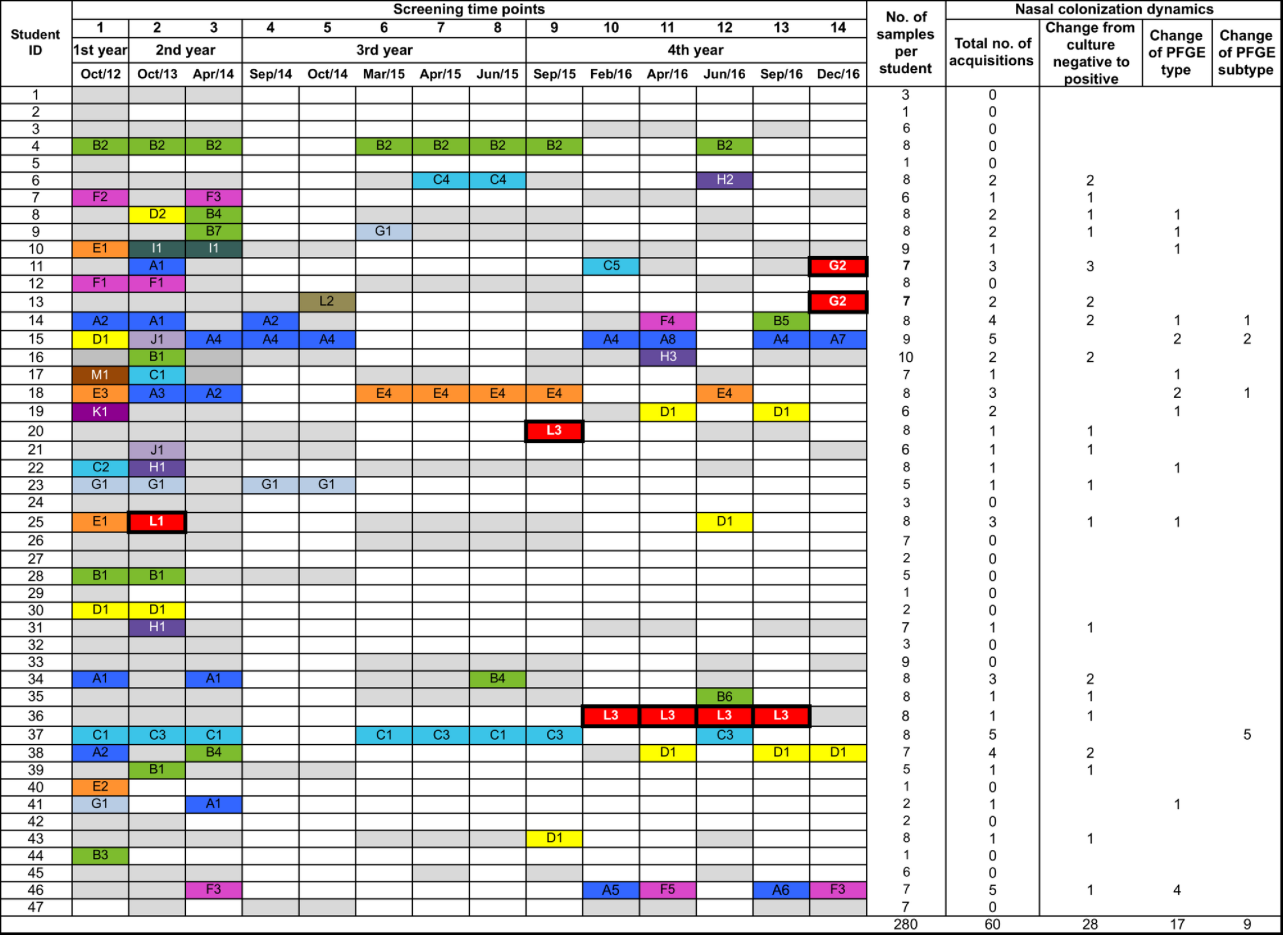
*S*. *aureus* population dynamics among nursing students over time. White cell = no screening; Grey cell = culture negative; Colored cell = culture positive (MSSA); Red cell = culture positive (MRSA).

A total of 20 students (43%) were nasally colonized with methicillin-susceptible *S*. *aureus* (MSSA) at admission, but none was colonized with MRSA–Table 1. However, throughout the four years of university attendance, 14 other students (30%) became colonized with *S*. *aureus*. Globally, a very high proportion of students (34 out of 47; 72%) were found to be *S*. *aureus* nasal carriers at some time during the study period (Fig 1).

**Table 1:** Identification of *Staphylococcus aureus*, including MRSA, among nursing students.

	1st year	2nd year	3rd year	4th year	Total
	(2012–13)	(2013–14)	(2014–15)	(2015–16)	(2012–16)
No. of screenings	1*	2	5	6	14
Individuals screened	46 (98%)	42 (89%)	27 (57%)	32 (68%)	47 (100%)
Female	39 (85%)	36 (86%)	21 (78%)	29 (91%)	40 (85%)
*S*. *aureus* nasal carriers	20 (43%)	25 (62%)	10 (37%)	17 (53%)	34 (72%)
No. of clonal types (MSSA)	9	8	6	7	13
Clonal types (MSSA) **	A-ST30, B-ST72, C-ST508, D-ST398, E-ST7, F-ST15, G-ST3162, K-ST34, M-ST27	A-ST30, B-ST72, D-ST398, F-ST15, G-ST3162, H-ST97, I-ST10, J-ST8	A-ST30, B-ST72, C-ST508, E-ST7, G-ST3162, L-ST22	A-ST30, B-ST72, C-ST508, D-ST398, E-ST7, F-ST15, H-ST97	A-ST30, B-ST72, C-ST508, D-ST398, E-ST7, F-ST15, G-ST3162, H-ST97, I-ST10, J-ST8, K-ST34, L-ST22, M-ST27
MRSA nasal carriers	0	1 (2%)	0	4 (13%)	5 (11%)
No. of clonal types (MRSA)		1		2	2
Clonal types (MRSA) ***		L-ST22-IVh		L-ST22-IVh, G-ST3162-II	L-ST22-IVh, G-ST3162-II
Students exposure to nursing homes (hours)	0	120	0	0	120
Students exposure to hospital environment (hours)	0	0	450	630	1080
Students exposure to ICU, emergency, surgery room (hours)	0	0	0	260	260

*At university admission (October 2012)

**Defined by pulsed-field gel electrophoresis and multilocus sequence type; Underlined clonal types are present in the four years

*** Defined by pulsed-field gel electrophoresis, multilocus sequence type, and staphylococcal chromosomal cassette *mec* typing.

MSSA—Methicillin-susceptible *Staphylococcus aureus*; MRSA—Methicillin-resistant *S*. *aureus*; ST—Sequence type; ICU—Intensive care unit

Among the different variables analyzed, having contact with animals and being a smoker were significantly associated with *S*. *aureus* nasal colonization (p = 0.005 and p = 0.001, respectively). None of the variables was identified as a protective factor for nasal colonization.

Among the participating students, 26 (55%) were classified as intermittent carriers, four (9%) as persistent carriers and nine (19%) as non-carriers. Only one persistent carrier and three intermittent carriers harbored the same strain (same PFGE subtype/*spa* type) at the different screening time points showing that *S*. *aureus* nasal carriage was highly dynamic among this population. Moreover, over the four-years of the study, we detected 60 *S*. *aureus* acquisitions involving 28 (60%) students. These 60 acquisitions corresponded to 28 cases of change of colonization status from culture negative to culture positive, 17 cases of change of PFGE type, and 9 cases of change of PFGE subtype in the same carrier (Fig 1).

The molecular characterization distributed the 97 methicillin-susceptible *S*. *aureus* (MSSA) isolates (recovered from the 47 individuals and 14 screening time points) into 13 clonal types (Table 2). Four clones included more than half (65%) of the isolates: A-ST30 (21%), B-ST72 (20%), C-ST508 (13%), and D-ST398 (11%). Clonal types A-ST30 and B-ST72 were recovered throughout the four years (Table 1). ST30 was the major MSSA clone in Portugal between 1992 and 2011 in both the hospital and the community [18,19], while clonal types ST72 and ST398 were only detected after 2001 [19].

**Table 2:** Characteristics of methicillin-susceptible *Staphylococcus aureus* recovered from nursing students.

PFGE type	No. of PFGE subtypes	*spa* type	ST	no. of isolates	%
A	8	t012, t018, t021, t136, t840	30	20	21%
B	7	t148, t1346, t3169, t14523	72	19	20%
C	5	t015, t130, t861, t6038	508	13	13%
D	1	t571, t13456	398	11	11%
E	4	t091, t114, t693	7	9	9%
F	5	t084, t279, t7523	15	8	8%
G	1	t002	3162	6	6%
H	3	t160, t267	97	4	4%
I	1	t166	10	2	2%
J	1	t024	8	2	2%
K	1	t166	34	1	1%
L	1	t14524	22	1	1%
M	1	t100	27	1	1%

PFGE–Pulsed-field gel electrophoresis; ST–sequence type.

Noteworthy, none of the students carried MRSA at the admittance of the nursing school but five (11%) became colonized during the university attendance. One student became colonized in the second year of studies while four students were colonized in the last year, presumably as a consequence of the increased exposure to the hospital environment, namely in intensive care units, emergency rooms and surgery wards during the clinical training (Table 1). None of these students had risk factors for MRSA carriage, with the exception of one individual whose mother worked in a nursing home but in another city.

The majority of the isolates were resistant to penicillin (75%). Resistance to clindamycin (20%), erythromycin (20%), cefoxitin (11%), ciprofloxacin (10%), fusidic acid (4%), rifampin (3%) and tetracycline (3% each) was also observed. Three isolates showed intermediate resistance to quinupristin-dalfopristin. None of the isolates was resistant to chloramphenicol, gentamicin, linezolid, mupirocin, teicoplanin, trimethoprim-sulfamethoxazole and vancomycin. All isolates were PVL negative.

## Discussion

In this longitudinal study, we evaluated *S*. *aureus* and MRSA nasal carriage among nursing students covering the four years of university attendance. The *S*. *aureus* colonization rate at the admittance (43%) was comparable to the one estimated in the general population (37%)[2]. However, the number of students that were colonized at some point during the four years increased considerably (72%). This value was much higher than the rates reported among medical students in other European countries, namely in France (27%) [20], Spain (35%) [21], Austria (25%) [22], and Bosnia (11%) [23] or in other continents, namely in the USA (16%) [24], Malaysia, Taiwan and Japan (10–36%) [25–27] and Colombia (25%) [28]. These differences may be due to the fact that the present study included a considerably higher number of screening time points (n = 14) compared to the previous studies; most were cross-sectional and therefore performed at a single screening point and the few prospective longitudinal studies usually evaluated the nasal carriage up to three time points.

Also higher rates of MRSA carriage were found in the present study (11%) compared to previous reports on medical or nursing students (0–5.4%), with the exception of an Israeli study that found up to 10% of the students harboring MRSA [8]. Possible explanations for these discrepancies may be the number of sampling times, the national MRSA prevalence, and the size of the sample. Portugal is a country with a very high MRSA prevalence (47%), one of the highest in Europe [12], which may increase the possibility of MRSA acquisition in the healthcare environment. This seems to be corroborated by the fact that MRSA colonization of the five Portuguese nursing students occurred after healthcare exposure. Moreover, three of the five MRSA carriers were colonized with the predominant MRSA clone circulating in Portuguese hospitals (ST22-IVh) [29], while the other two students carried ST3162-II. Noteworthy, this is the first report of ST3162, which is a single locus variant of ST5. ST5-II, previously designated as the New York/Japan clone [30], was introduced in Portuguese hospitals in 2005 [31] becoming the major clone in most of the hospitals in Lisbon in 2006 [32]. Notably, MSSA-ST3162 was detected in four students in the present study and therefore MRSA-ST3162-II might have originated by the introduction of SCC*mec* type II in an existing MSSA background.

Quinolones are often used in Portugal for the treatment of staphylococcal osteomyelitis for its good bone penetration and erythromycin is one of the choices in case of allergy to penicillin. The fact that 20% of the isolates colonizing the students showed resistance to both antibiotics may lead to a reduced therapeutic choice in case of an eventual future infection caused by *S*. *aureus*.

Our study showed increasing rates of *S*. *aureus* and MRSA nasal colonization among nursing students with increasing clinical exposure. Similar observations have been reported among medical students in Turkey [33]. Notably, nursing students are occupationally exposed to pathogens that may threaten their own health. It is known that *S*. *aureus* nasal carriage is an important risk factor for infection in individuals undergoing surgery [2] and therefore colonized nursing students became themselves more vulnerable. In addition, students colonized with MRSA will constitute potential transmission vectors of nosocomial strains to the community.

Our study presented some limitations as follows. First, none of the students was screened in all 14 time points, which might have underestimated the colonization rates with *S*. *aureus* and MRSA. Second, the inclusion of a control group of non-healthcare students was missing; this sampling could have corroborated that *S*. *aureus*, especially MRSA nasal colonization in nursing students was a consequence of clinical exposure during their nursing studies.

In summary, we reported very high rates of *S*. *aureus* (72%) and MRSA (11%) nasal carriage among nursing students in a Portuguese university. A considerable proportion of students became colonized by *S*. *aureus* (30%) including MRSA (11%) during the nursing education, mainly after clinical exposure. Colonization of nursing students was highly dynamic with continuous ingress of strains with distinct PFGE types or subtypes in the same individual. Three out of five MRSA isolates belonged to the prevalent clone (EMRSA-15, ST22-IVh) circulating in national hospitals. The results evidence that nursing students represent important reservoirs and vectors of *S*. *aureus*/MRSA inside hospitals and to the community. Therefore, education on infection control measures in nursing schools is of major importance as well as implementation of adequate and effective infection control programs to reduce the extremely high prevalence of MRSA in Portuguese hospitals that remains over 45% [12].
